# Repetitive transcranial magnetic stimulation for fibromyalgia: are we there yet?

**DOI:** 10.1097/PR9.0000000000001221

**Published:** 2025-01-13

**Authors:** Jorge Dornellys da Silva Lapa, Valquíria Aparecida da Silva, Daniel Ciampi de Andrade

**Affiliations:** a Neurosurgery Unit, Hospital de Cirurgia, Aracaju, Sergipe, Brazil; b Department of Medicine, Federal University of Sergipe, Aracaju, Sergipe, Brazil; c Service of Interdisciplinary Neuromodulation (SIN), Department and Institute of Psychiatry, University of São Paulo Medical School, São Paulo, Brazil; d School of Nursing, Medical-Surgical Nursing Department, University of São Paulo, São Paulo, SP, Brazil; e Center for Neuroplasticity and Pain (CNAP), Department of Health Science and Technology, Faculty of Medicine, Aalborg University, Aalborg, Denmark

**Keywords:** Fibromyalgia, Pain, Transcranial magnetic stimulation

## Abstract

Repetitive transcranial magnetic stimulation (rTMS) has increasingly been used to modify cortical maladaptive plastic changes shown to occur in fibromyalgia (FM) and to correlate with symptoms. Evidence for its efficacy is currently inconclusive, mainly due to heterogeneity of stimulation parameters used in trials available to date. Here, we reviewed the current evidence on the use of rTMS for FM control in the format of a narrative review, in which a systematic dissection of the different stimulation parameters would be possible. We conducted a search in Medline and Embase for controlled trials on rTMS in people with FM with at least 10 participants in each treatment arm, and treatment/follow-up of at least 3 weeks. The search identified 482 abstracts, of which 45 were screened to full review, and 11 met inclusion criteria. Six out of 11 trials were positive. The dorsolateral prefrontal cortex was the target in 218 patients (49.2%), and the primary motor cortex (M1) in 225 (50.8%). Studies targeting M1 at 10 Hz, with stimulation current delivered in the posterior-anterior, were systematically positive, frequently showing that maintenance sessions delivered weekly, and biweekly were able to maintain the analgesic effects seen after daily induction sessions. Studies assessing the effects of rTMS for FM are still marked by heterogeneity in stimulation petameters, choice of primary outcomes, and inclusion criteria. The selection of the stimulation parameters associated with significant analgesic effects is likely to benefit following larger multicenter trials and improve the overall management of pain and associated symptoms in people with FM.

## Abstract 2

Supplemental Digital Content is Available in the Text.

Repetitive transcranial magnetic stimulation has been used to treat fibromyalgia in several randomized controlled trials. Its clinical effects seem to depend on specific stimulation parameters.

## 1. Introduction

Fibromyalgia (FM) is a primary chronic pain disorder.^42^ Nociplastic pain mechanisms are believed to play a significant role in the generation and maintenance of pain and related symptoms.^2,6,8,24,25^ Fibromyalgia prevalence has increased worldwide during the last years^40^ and is estimated to reach 2.7% in the general population.^31,39^ People with FM have a general lower quality of life, consume higher health care resources, and face lower work productivity compared to general population. These factors are, in part, due to the low efficacy of current therapeutic options^30,41^ and the subsequent impact of persisting symptoms on the lives of patients.^25,26,45^

More than a third of people with FM may not reach meaningful pain relief with medications and nonpharmacologic therapies are frequently offered as an attempt to improve clinical results.^41^ Among nonpharmacologic interventions, noninvasive brain stimulation techniques have increasingly been used to treat pain syndromes and other neuropsychiatric disorders.^18,28,29^ The most used technique is repetitive transcranial magnetic stimulation (rTMS).^17,19,22^ Repetitive transcranial magnetic stimulation is based on the principle of electromagnetic induction and refers to the application of a coil to the scalp, over the orthogonal projection of the cortical area that is being aimed at, and is believed to influence cortical maladaptive neuroplastic processes related to abnormal gain in pain processing^2,5,6,24,25^ in FM. Depending on several parameters, including frequency of the pulses delivered, depolarization of neuronal assembles may occur, and long-term plastic changes in cortical activity can be induced.^35,47^ Repetitive transcranial magnetic stimulation delivered to the dorsolateral prefrontal cortex (dlPFC) at high frequency (usually above 10 Hz) or to the right dlPFC at low frequencies (ie, 1 Hz) is approved by the FDA for the management of major depression,^11^ and its use over the motor cortex (M1) has been repetitively shown to control neuropathic pain in larger trials,^3,4^ and lately, for the first time, it was formally recommended in national guidelines for the control of neuropathic pain.^36^ Stimulation to the dlPFC and the motor cortex are known to induce long-lasting analgesic effects in healthy people,^37^ and to increase pain thresholds toward analgesia, which depends on the availability of N-methyl-d-aspartate glutamate receptors to occur.^10,35^ Animal,^38^ healthy human,^13^ and patient studies^32^ have shown that M1 stimulation has part of its effects depending on the release of endogenous opioids, while evidence for this in dlPFC rTMS is less clear. People with fibromyalgia have been shown to have defective GABA-dependent intracortical inhibition, which is correlated to symptoms severity, and which is restored toward normal values under treatment with motor cortex rTMS.^34,35^

Despite the mechanistic framework supporting the analgesic effects of rTMS for FM, studies targeting M1 or dlPFC have shown conflicting results. While the heterogeneity of these studies precludes quantitative literature synthesis analyses, we believe a concise narrative review would allow for the dissection of the most important variables leading to the current conflicting results and pave the way for future trials in the field.

## 2. Methods

A search on Medline (through PubMed) and Embase from inception to February 2024 with the following research strategy was done: (“Noninvasive brain stimulation”OR“Transcranial magnetic stimulation”)AND(“Fibromyalgia” OR “Fibromyalgia syndrome”). Reference and citation list of relevant studies were screened for eligibility: (1) prospective and controlled trials; (2) reporting fibromyalgia diagnosis based on American College of Rheumatology and with pain as the main outcome; (3) use of repetitive transcranial magnetic stimulation; (4) at least 10 participants in each treatment arm; and (5) treatment/follow-up of at least 3 weeks. Articles were searched by J.D.S.L. and D.C.A. and data extracted by J.D.S.L. and V.A.S. J.D.S.L., D.C.A., and V.A.S. analyzed and tabulated the included studies. In the absence of sufficient quantitative data for a meta-analysis, a qualitative presentation of the data has been made in the form of a narrative review.

## 3. Results

Search retrieved 143 and 339 abstracts in Medline and Embase, respectively. We reviewed 482 abstracts, 45 full articles were selected for review, and 11 meeting inclusion criteria (n = 443 women). Figure S1, http://links.lww.com/PR9/A271 summarized the steps of literature search and study selection. The dlPFC was the target in 218 patients (49.2%), and the primary motor cortex (M1) in 225 (50.8%). Data from dlPFC TMS were retrieved from 6 studies, including one study including people with major depression and comorbid FM,^6,9,16,42,43,46^ while data from M1 TMS were retrieved from 5 other studies.^7,20,27,33,39^ Six out of 11 trials were positive. Concerning the dlPFC target, one study (n = 28), included people with major depression comorbid with FM (negative study),^9^ while in 2 studies^40,46^ (n = 54), people with major depression were allowed to participate (both positive studies). In 3 studies (n = 136), people with major depression were excluded (2 negative studies and one positive study).^6,16,43^ In all M1 studies,^7,20,27,33,39^ major depression was an exclusion criterion (3 positive and 2 negative studies). The duration of studies was in average 13 weeks (range 4–25). The mean treatment duration was 9 weeks, and the mean follow-up post-treatment was 4 weeks. Full details of studies are in Tables S1 and S2, http://links.lww.com/PR9/A271.

## 4. Discussion

The primary outcome was reached in 6 out of 11 studies. Primary outcomes and outcome measures varied to a large extent and included diverse pain intensity measures^6,9,33,39,42,43,46^ percentage of responders defined with different methodologies,^27^ improvement assessed by the global impression of change,^27^ quality of life assessment,^7^ and the burden of FM symptoms.^16,20^ In some studies, the main outcomes were assessed after the induction treatment,^7,9,27,39,42,46^ in some after the maintenance period,^6,20^ and in some after a follow-up period without treatment.^16,33,43^

Beyond this heterogeneity lies the diversity of rTMS parameters used, starting by the stimulation target. The dlPFC and M1 are engaged differently during acute^14,15^ and chronic pain,^1^ and the underlying mechanisms of their analgesic effects are considered to be different.^12^ Regarding dlPFC targeting, the right side of stimulation at low frequency (1 Hz) showed more consistently positive results^41,44^ than the left side at high frequency^42^ (ie, 10 Hz). Both trials using low-intensity stimulation of the dlPFC were positive, except for one that included people with depression. Left side stimulation trials provided negative results,^6,16^ except for a positive trial^42^ with the lowest number of participants among all studies to date. It was noteworthy that even when neuronavigation was used, the definition and the way to identify the dlPFC varied enormously across studies and that may explain part of the heterogeneity in the findings.^6,16,42,46^ Results of M1 stimulation are also heterogeneous, but some interesting features can be identified across trials. Analyzing the 2 negative trials,^20,27^ both targeted the motor cortex via unusual^27^ or not referenced^20^ techniques. Also, the direction of the stimulation was not reported in these 2 trials.^20,27^ It has been shown in people with neuropathic pain that anterior-posterior TMS-induced current orientation can provide analgesic effects, while latero-medial one does not, being similar to sham.^1^ This is thought to be due to the differential types of fibers within M1 that are activated by different stimulation orientations. In addition, in one of the negative studies, rTMS was added to a standardized rehabilitation protocol^20^ received by all participants, which could insert ceiling effects. One of the negative studies reported negative results due to unsuccessful maintenance sessions where stimulation sessions occurred every 3 weeks. However, active rTMS was significantly superior to sham during the 3-week induction phase.^27^ Among the positive studies,^7,33,39^ all aimed at the motor hot spot (the representation on M1 of a specific muscle) based on neurophysiological and individual data and performed stimulations in the anterior-posterior direction over M1.

One important issue is the presence of major depression comorbid to FM. While mood symptoms are an inherent part of the FM syndrome, major depressive disorder affects only around 22% of people with FM.^23^ M1 stimulation has been shown to improve mood symptoms in patients with FM without major depression,^33^ but the effects of rTMS on both pain and major depression symptoms remains to be determined. Allowing a variable amount of people with major depression comorbid to FM in trials^42,46^ may add variability to these studies and may negatively affect their sensitivity to detect changes between treatment arms. One possibility would be to design specific trials for FM comorbid to major depressive disorder, as done previously,^9^ and accept the more restricted external validity of this approach. The interaction between pain and mood improvement can be rather complex. Indeed, it has been shown that antidepressant drugs having also analgesic effects provide analgesic and antidepressive effects that occur in different degrees across individuals, and in a differential temporal profile in those experiencing improvement in both domains.^21^

In conclusion, studies assessing the effects of rTMS for FM are still marked by heterogeneity in outcome and in the parameters of stimulation. The inclusion of people with comorbid major depression is a major point to consider where designing clinical trials, as well as the targeting technique. Low-frequency stimulation of the right dlPFC has shown promising results, as well as M1 stimulation based on the representation of the motor hot spot of hand muscles and with a current delivered in the anterior-posterior orientation. Larger multicenter and international trials accounting for these variables will possibly help to share light into the use of rTMS for pain relief in people with FM, along with integrative reviews including prestudy registry in repositories and formal assessment for risk of bias.

## Disclosures

The authors have no conflict of interest to declare.

## Supplemental digital content

Supplemental digital content associated with this article can be found online at http://links.lww.com/PR9/A271.
